# New Appliances and Things Medical

**Published:** 1900-03-10

**Authors:** 


### NEW APPLIANCES AND THINGS MEDICAL.

**[We shall be glad to receive, at our Office, 28 & 29, Southampton Street, Strand, London, W.C., from the manufacturers, specimens of all new preparations and appliances which may be brought out from time to time.]**

### CHICKEN BROTH JELLY AND BEEF TEA.

(C. Shippam, Chichester.)

The above invalid specialities in no way fall short of the high standard of excellence maintained for many years by the Chichester firm in all departments of table specialities. The chicken broth jelly is a delicately flavoured preparation of most careful manufacture. It may safely be relied upon as of genuine origin. The beef-tea is a preparation which is practically unique in our experience. It is exactly such as is turned out by the best cooks in private establishments where time and expense are no object. Beef-tea is frequently an emergency requirement. With Shippam's bottled variety at hand ready for immediate use the emergency can and should be instantly met. We believe that were this preparation better known to the medical profession it would be generally acknowledged that the interests of patients would be better studied by ordering this variety of beef-tea of constant and reliable preparation than the indifferent and unsatisfactory commodity which passes under the same name and which is the best that can be obtained in many households.

### THE MONTAGUE ROSE CREAM SOAP.

(Agents: Arthur & Co., 69, Berners Street, London, W.)

This is an excellent superfatted soap of emollient and antiseptic properties. It appears to possess a tonic and astringent action on the skin, and hence for toilet purposes it has the combined advantages of a first-rate soap and of a valuable medicament.

### PANOPEPTON.

(Fairchild Brothers and Foster, New York, U.S.A. London Agents: Burroughs, Wellcome, and Co., Snow Hill, London, E.C.)

This is a ready-made peptonised food, which, for invalids such as require a predigested dietary, is probably superior in nearly all respects to similar foods, the result of home peptonisation. The process of digestion is carried out in a scientific manner and under the most experienced direction. Panopepton is prepared from lean beef and wheat flour, thus combining the advantages of both vegetable and animal food. It is exceedingly palatable, and, being immediately absorbed on entering the stomach, it acts as an instantaneous restorative and is valuable in cases of emergency, or in cases where gastric digestion is in abeyance.

